# Friend virus limits adaptive cellular immune responses by imprinting a maturation-resistant and T helper type 2-biased immunophenotype in dendritic cells

**DOI:** 10.1371/journal.pone.0192541

**Published:** 2018-02-09

**Authors:** Limei Shen, Stefan Tenzer, Moritz Hess, Ute Distler, Ingrid Tubbe, Evelyn Montermann, Simone Schimmer, Ulf Dittmer, Stephan Grabbe, Matthias Bros

**Affiliations:** 1 Department of Dermatology, University Medical Center, Mainz, Germany; 2 Institute of Immunology, University Medical Center, Mainz, Germany; 3 Institute for Medical Biometry, Epidemiology and Informatics, University Medical Center, Mainz, Germany; 4 Institute for Virology of the University Hospital Essen, University of Duisburg-Essen, Essen, Germany; University of Colorado Denver, UNITED STATES

## Abstract

The murine Friend virus (FV) retrovirus model has been widely used to study anti-viral immune responses, and virus-induced cancer. Here we analyzed FV immune evasion mechanisms on the level of dendritic cells (DC) essential for the induction of primary adaptive immune responses. Comparative quantitative proteome analysis of FV-infected DC (FV-DC) of different genotypes (BALB/c, C57BL/6) and non-infected DC revealed numerous genotype-independently regulated proteins rergulating metabolic activity, cytoskeletal rearrangements, and antigen processing/presentation. These alterations may promote virion production in FV-DC. Stimulation of FV-DC with LPS resulted in strongly enhanced IL-10 production which was partially responsible for their attenuated T cell (CD4^+^, CD8^+^) stimulatory capacity. Stimulated FV-DC induced less IFN-γ production in T cells required for cellular anti-viral responses, but more T helper cell type 2 (Th2)-associated cytokines (IL-4, IL-5, IL-13). We conclude that FV reprograms DC to promote viral spreading and immune deviation by imprinting a largely maturation-resistant, Th2-biased immunophenotype.

## Introduction

The murine Friend virus (FV) infection model has been widely used to dissect the role and interplay of anti-retroviral innate [1] and adaptive [2, 3] immune responses. FV constitutes a gamma-retrovirus complex composed of the pathogenic, but replication-defective SFFV (spleen focus-forming virus) and replication-competent F-MuLV (Friend murine leukemia virus), and readily infects hematopoietic progenitors but also differentiated immune cells [4–6]. FV infection takes very different courses in various mouse strains: whereas BALB/c mice rapidly develop fatal erythroleukemia and succumb to the disease, C57BL/6 mice control the infection resulting in chronic asymptomatic virus persistence [7]. Strain-associated differences in FV susceptibility and disease progression have been attributed to functional allelic differences in Apobec-3 expression which limits viral propagation at early stages of infection in resistant, but less so in susceptible mice [8]. In addition, BALB/c but not C57BL/6 erythroblasts express a short isoform of the Stk kinase which interacts with a SFFV envelope protein (gp55) to promote erythroblast expansion [9].

Besides the decisive role of innate anti-viral defense molecules like Apobec-3 [10] for early control of FV spread, the disease course is also affected by differential immune recognition and responses to FV. In this regard, FV-susceptible BALB/c mice were shown to lack the ability to present immunodominant FV antigens to CD8^+^ T cells due to their MHCI haplotype, in contrast to FV-resistant C57BL/6 mice [11]. Moreover, we and others have shown that FV-specific IFN- γ producing CD4^+^ type 1 T helper (Th) [12] and cytotoxic CD8^+^ T cells (CTL) [13] are important in C57BL/6 mice to limit FV infection. The induction of Th1 cells during acute infection is followed by the expansion of CD4^+^ regulatory T cells in FV-resistant mice (Treg) [2, 14]. These Tregs impair both Th1 and CTL effector functions [15, 16]. Furthermore, during FV infection, antigen-specific CD8^+^ T cells express inhibitory receptors (i.e., PD-1, Tim-3, LAG-3, and CTLA-4), and acquire a dysfunctional state during chronic infection [6]. Similarly, B cells also contribute to prevent disease aggravation in C57BL/6 mice, since depletion of B cells strongly impaired the survival of FV-resistant mice [17]. Altogether, a number of studies have demonstrated that FV-specific T cells and B cells are essential to limit the extent of FV infection in resistant mice, but this response is counter-regulated by FV-induced Tregs.

Whereas the relevance of these adaptive immune responses for the control of FV infection has been addressed in a number of studies, potential direct regulatory effects of FV on dendritic cells (DC) as the main inducers of pathogen-specific cellular and humoral immune responses [18] have not been thoroughly analyzed yet. We have previously shown that FV-infected DC from BALB/c mice were characterized by reduced expression of costimulatory surface molecules, and accordingly induced less effector CD4^+^ T cell stimulation, but expanded CD4^+^ Tregs [16].

Here we asked whether FV infection also affected DC of C57BL/6 background as observed by us for BALB/c DC. Therefore, we compared the immuno-phenotype and the T cell stimulatory and polarizing capacitiy of FV-infected and non-infected DC of either genotype. To elucidate the impact of FV infection on DC biology on a molecular level, we also performed an unbiased proteome analysis.

Besides a number of proteins regulated by FV in a genotype-specific manner, we also identified many proteins that were commonly regulated in FV-infected bone marrow derived (BM-) DC (FV-DC) of either genotype. Network analysis showed that FV-infected DC commonly regulated proteins involved in metabolism and cytoskeletal regulation compared to non-infected DC. Moreover, MHC-II and costimulatory molecule expression was strongly attenuated in infected DC, resulting in a reduced capacity to stimulate T cell proliferation. These effects were at least in part mediated by FV induced IL-10, as shown by using FV-infected IL-10^-/-^ BM-DC. Furthermore, in a genotype-independent manner FV-DC induced lower production of CD4^+^ Th1 and CD8^+^ T cell type 1 (Tc1)-associated IFN- γ required for eliminating FV-infected cells, but higher amounts of the Th2 cytokines IL-4, IL-5, and IL-13. This Th2-bias in cytokine production may contribute to FV immune evasion and establishment of chronic infection.

## Materials and methods

### Friend retrovirus

The Friend virus (FV) stock used for all infections contained the B-tropic F-MuLV and SFFV, was devoid of LDV, and was generated as described [19]. Mice were infected by i.v. inoculation via the tail vein. To overcome the resistant state of C57BL/6 mice towards FV infection, a higher dose (10,000 IU) was applied as compared with BALB/c mice (3,000 IU).

### Mice

All mouse strains used (BALB/cByJ, C57BL/6J, DO11.10, OT-I, OT-II, CD45.1xC57BL/6J, IL-10^-/-^ C57BL/6J) were bred and maintained in the Central Animal Facilities of the University of Mainz under specific pathogen-free conditions on a standard diet. The recommendations of the Guide for the Care and Use of Laboratory Animals by the National Institutes of Health (NIH Publications No. 8023, revised 1978) were followed. The Ethics Commission according to the German Animal Welfare Act (Landesuntersuchungsamt of the state Rhineland-Palatinate) approved the experiments in this study (Reference no. Az. 23 177-07/G 09-1-033). Mice were euthanized by carbon dioxide asphyxiation. CD4^+^ T cells of DO11.10 mice (BALB/c background) [20] and OT-II mice (C57BL/6 background) [21] express a transgenic T-cell receptor (TCR) which recognizes ovalbumin (OVA) peptide 323–339 (ISQAVHAAHAEINEAGR) in the context of H-2 I-A^d^ (BALB/c) and I-A^b^ (C57BL/6), respectively. CD8^+^ T cells of OT-I (C57BL/6) mice are transgenic for a αβTCR specific for OVA_257-264_ peptide in the context of H-2Kb [22]. DO11.10, OT-I and OT-II mice were crossed with CD45.1^+^ C57BL/6J congenic mice.

### Reagents

eFluor-conjugated anti-MHC-II, APC-labeled anti-CD40, FITC-conjugated anti-CD80, anti-CD86, and anti-CD4, APC-Cy7-conjugated anti-CD8, PE-conjugated anti-Vα2 and anti-CD25, PE-Cy5-conjugated CD45.1, PE-Cy-7-, BV-421-conjugated anti-CD11c, and APC-conjugated anti-IFN- γ antibodies were purchased from eBioscience (San Diego, CA). CFSE (carboxyfluorescein diacetate succinimidyl ester) was purchased from Life Technologies (Carlsbad, CA).Immunostimulatory CpG1826 was obtained from Miltenyi Biotech (Bergisch Gladbach, Germany), and LPS from Calbiochem (San Diego, CA).

### DC cultures

One week after FV infection, FV-infected splenic DC and FV-infected bone marrow (BM) cells were sorted using a PE-labeled mouse antibody that reacts with F-MuLV-encoded host cell surface protein Glyco-Gag (Ab34) [23]. Bone marrow-derived DC (BM-DC) from non-infected mice or p34^+^ BM-DC from FV-infected mice were generated as previously described [16] with some modifications. In brief, 3x10^6^ BM cells were resuspended in 4 ml of RPMI 1640 medium (PAA Laboratories, Pashing, Austria), supplemented with 5% FCS, 2 mM L-Glutamine, 0.1 mM nonessential animo acids, 50 μg/ml gentamycin (all from PAA), 50 μM β-mercaptoethanol (Sigma-Aldrich, Deisenhofen, Germany), and 4 ng/ml recombinant murine GM-CSF (R&D, Wiesbaden, Germany). BM cells were seeded into 6-well cell culture plates (BD, Franklin Lakes, NJ). On day 3 of culture, fresh medium (4 ml) was added. On day 6, non-adherent and loosely adherent BM-DC were harvested, washed and subjected to experiments. For stimulation, LPS (100 ng/ml) was added overnight. Splenic DC were activated with CpG1826 (500 ng/ml) since this stimulus yielded stronger upregulation of DC surface markers than LPS (not shown).

### Flow cytometry

Cells were washed in FACS buffer (PBS, 1% FCS, 0.5 mM EDTA), and were stained with antibodies as indicated. Intracellular IFN- γ expression of OT-I and OT-II T cells was detected as described [24]. Expression intensities were assessed by flow cytometry (LSR II, BD) and data were analyzed using FACSDiva software (V 8.0.1; BD). The gating strategy is depicted in S1 Fig.

### Multiplex Cytometric Bead (CBA) Assay

Cytokine contents in supernatants of stimulated splenic DC and BM-DC, and BM-DC/T cell cocultures (see below) were detected by CBA (BD). Briefly, bead populations with distinct fluorescence intensities were conjugated with cytokine-specific capture antibodies. Recombinant cytokines and cell culture supernatants were mixed with the bead populations. Samples were incubated with PE-conjugated detection antibodies, followed by analysis in the FL3 channel of a LSR-II flow cytometer (BD). Results were analyzed using CBA Analysis Software (BD).

### In vitro T cell proliferation

On day 6 of culture, BM-DC (0.75x10^6^ cells) were incubated with OVA protein (10 μg/ml). After 3h, aliquots were stimulated with LPS (100 ng/ml). On the next day, BM-DC were harvested and thoroughly washed. CD4^+^ (DO11.10, OT-II) or CD8^+^ (OT-I) T cells were isolated from mouse spleens and lymph nodes (LNs) by immuno-magnetic separation (autoMACS Pro Separator, Miltenyi Biotec). BM-DC (5x10^4^) were cocultured with syngeneic OVA peptide-reactive T cells (each 10^4^) in triplicates for 96 h in 96-well cell culture plates (BD). T cell proliferation was assessed by detecting incorporation of ^3^H-thymidine during the last 16 h of coculture in a ß counter (1205 Betaplate, LKB Wallac, Turku, Finnland).

### In vivo T cell proliferation

Splenocytes derived from mice with transgenic T cell receptors (DO11.10xCD45.1, OT-IxCD45.1, OT-IIxCD45.1) were labelled with 0.5 μM CFSE for 10 min. CFSE-labeled splenocytes (10^7^ in 200 μl PBS) were transferred i.v. via the tail vein into C57BL/6 mice. After 48 h, BM-DC loaded with OVA peptide (1 μg/ml) and stimulated with LPS (100 ng/ml) were injected (3x10^6^ BM-DC/mouse). Three days later, spleens and peripheral LN were removed and pooled, and cell suspensions were analyzed for proliferation and frequencies of IFN- γ^+^ CFSE-labeled T cells by flow cytometry as described [24].

### Label-free quantitative proteomic analysis

On day 6 of culture BM-DC populations (Ctrl-DC, FV-DC) of either genotype (BALB/c, C57BL/6) were harvested, and label-free quantitative proteomic analyses of cytoplasmic proteins by mass spectrometry using ion-mobility enhanced data-independent acquisition were performed as described in detail previously [25]. Comparative proteome analysis was performed in five technical replicates.

### Bioinformatics and statistical analysis

Quantified protein abundances per gene were quantile-normalized and log transformed. Afterwards, Limma software [26] was employed to infer differential protein abundance between FV-infected and untreated cells. Only proteins that had a significant False Discovery Rate (FDR) of < 0.01 for treatment main effect and no significant (FDR< 0.01) interaction effect of strain and treatment were retained for the subsequent analysis. Gene Ontology (GO) overrepresentation analysis was performed with the retained significant proteins using topGO (https://bioconductor.org/packages/release/bioc/html/topGO.html) [27]. All analyses were performed in the statistical programming environment R (http://www.R-project.org) [28] using Bioconductor routines [29]. Potential interaction of proteins identified as differentially regulated in FV-DC were analyzed using the STRING database v10 (http://string-db.org) [30]. The probability of protein interaction correlates with the thickness of connecting lines (confidence view). Statistical analysis was performed using GraphPad Prism Software, 4.0 (Graph Pad Software Inc., San Diego, CA). Results were expressed as mean ± standard error of mean (SEM). Differences among groups were tested by ANOVA. Data were normally distributed and the variance between groups was not significantly different. Differences between two groups were tested using the paired Student’s t-test, assuming significance at *P* < 0.05.

## Results

### FV infection alters the proteome of DC

We have previously demonstrated that FV infected BALB/c BMDC displayed a largely maturation-resistant immune-phenotype and were poor T cell stimulators [16]. To gain broader insight into FV-regulated alterations of DC biology we assessed the proteome of FV-infected versus non-infected (Ctrl) DC of BALB/c mice, and also included FV-infected DC derived from C57BL/6 mice into our analysis to identify proteins that are regulated either in a synchronous fashion or differentially by infection in both mouse strains.

**Fig 1:**
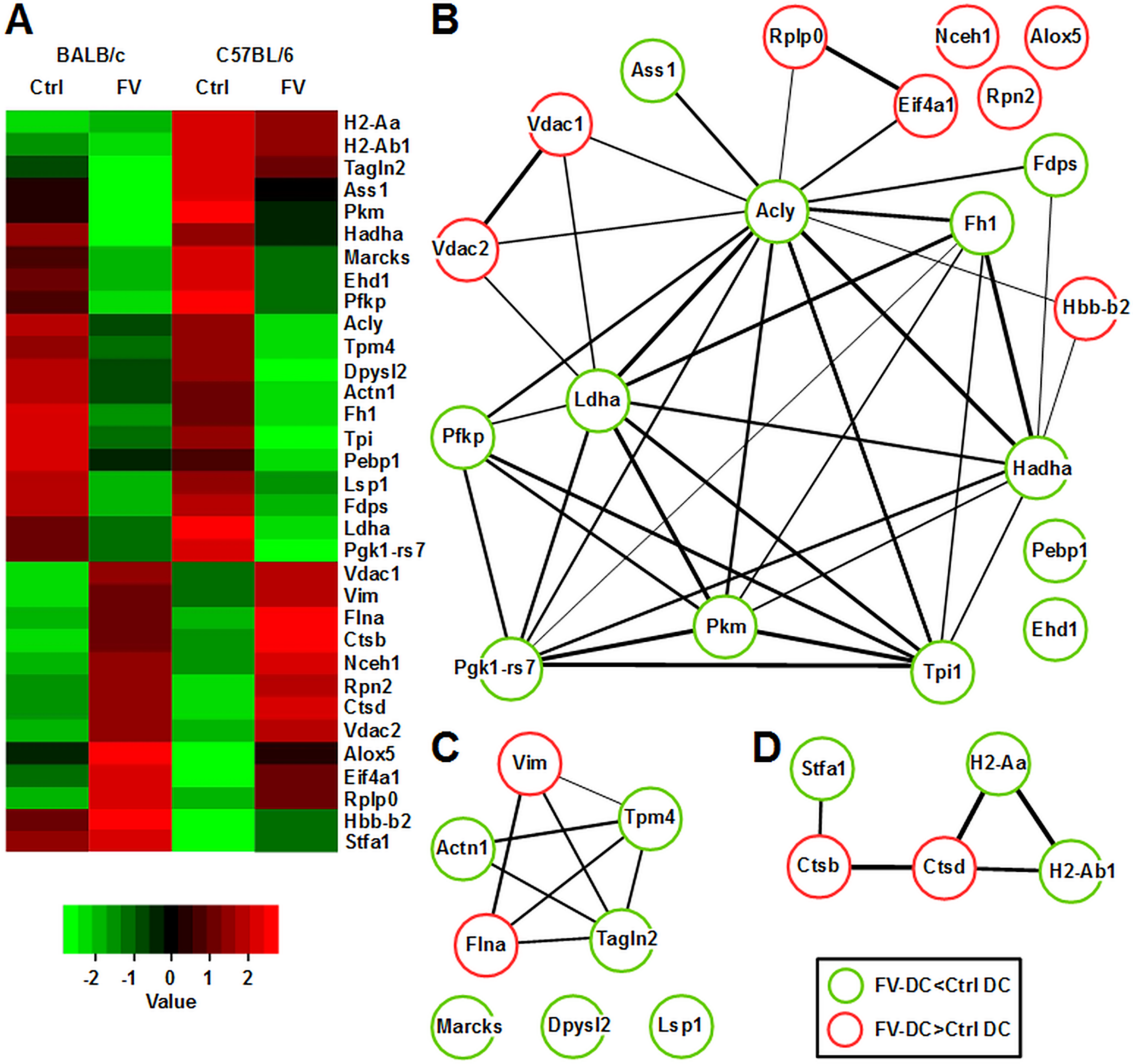
FV-DC from mice with different H-2 genotypes show converging FV-induced expression of groups of interacting proteins that regulate key cell functions. FV-DC were generated as described. On day 6 of culture, cytoplasmic protein was extracted form immature BM-DC (uninfected and FV-infected) and was subjected to label-free protein mass spectroscopy. (A) Heat map of cytoplasmic proteins concurrently regulated in FV-DC versus Ctrl-DC in a genotype-independent manner (see S1 File). (B-D) Subsets of proteins that are regulated in a concurrent fashion in both mouse strains (as shown in A) were grouped according to their primary function, namely (B) metabolism, (C) cytoskeletal organization, and (D) MHC-II biology. (B-D) The probability of protein-protein interaction correlates with the thickness of their connection lines.

To obtain sufficient numbers of FV-infected C57BL/6 DC, mice of this genotype were infected with much higher numbers of infectious FV particles as compared with BALB/c mice. One week after infection, the spleens of C57BL/6 mice contained lower frequencies of FV-infected CD11c^+^ DC than those of infected BALB/c mice, but FV-infected DC were readily detectable in both mouse strains (S2A Fig). Similarly, FV infected more BM cells in BALB/c than in C57BL/6 mice, (S2B Fig). FV-infected BM cells were immune-magnetically sorted and differentiated to conventional DC (BMDC). Then, comparative mass spectroscopy of cytoplasmic proteins derived from sorted FV-DC and and non-infected control DC (Ctrl) at an unstimulated (“immature”) state was performed. By this approach, we identified a large group of FV-regulated proteins (>300; S1 File). Besides several proteins that were differentially regulated by FV infection in a genotype-dependent manner, we observed that a number of proteins was congruently regulated in FV-DC of either genotype as compared with the corresponding non-infected Ctrl DC population (Fig 1A). We focussed on this group of FV-regulated proteins to identify common FV-induced protein network alterations.

We observed FV-induced coordinated regulation of a larger number of proteins that control cell metabolism, and a number of these was reported to form a network of interaction (Fig 1B). Enzymes involved in glucose metabolism, namely glycolysis (Pgk1-rs7 [phosphoglycerate kinase-1, related sequence-7],Pfkp [phosphofructokinase C], Tpi1 [triosephosphate isomerase 1], Pkm [pyruvate kinase]), conversion of pyruvate to lactate (Ldha [lactate dehydrogenase A]), and the mitochondrial citric acid cycle (Fh1 [fumarate hydratase 1], Hadha [hydroxyacyl-Coenzyme A dehydrogenase]) were downregulated as compared with Ctrl DC. Gene set enrichment analysis largely confirmed down-regulation of these metabolic processes in FV-DC (S2 File). Enzymes that link carbohydrate metabolism and fatty acid synthesis (Acly [ATP citrate lyase]), mediate protein glycosylation (Rpn2 [ribophorin II]), and are involved in the urea cycle (Ass1 [argininosuccinate synthetase 1]) were apparent at lower levels in FV-DC as well. Furthermore, FV-DC may contain lower levels of cholesterol due to lower contents of Fdps [farnesyl diphosphate synthetase] implicated in cholesterol synthesis and of Ehd1 [EH-domain containing 1] required for intracellular cholesterol storage. In line, levels of Nceh1 that hydrolyzes cholesterol were elevated in FV-DC. Moreover, FV-DC displayed elevated levels of Alox5 [arachidonate 5-lipoxygenase] which catalyzes the first step in leukotriene synthesis. In FV-DC, we also observed enhanced levels of anion-regulated channels (Vdac [voltage-dependent anion channel] 1, 2) that facilitate metabolite trafficking across the mitochondrial membrane, and of the hemoglobin beta adult minor chain (Hbb-b2) reported as expressed also by myeloid immune cells and shown to interact with several mitochondiral proteins including ATP synthase subunits. In addition, ribosomal proteins as required for mRNA translation (Eif4a1 [eukaryotic translation initiation factor 4A1], Rplp0 [ribosomal protein, large, P0]) were enhanced in FV-DC as well. Altogether, FV-induced alterations of the DC metabolism may reflect FV virion production.

Proteomic analysis also revealed FV-induced alterations in the levels of several cytoskeletal proteins, and several of these were predicted to interact (Fig 1C). Of these cytoskeletal regulators, only Vim (vimentin) which forms intermediate filaments displayed elevated expression in FV-DC as compared with Ctrl DC. In contrast, actin-binding proteins, including Tpm4 (tropomyosin 4), Tagln2 (transgelin 2), Flna (filamin, alpha), Actn1 (actinin, alpha 1), and Myh10 (myosin, heavy polypeptide 10) were expressed at lower levels. We also observed attenuated expression of the cytoskeletal regulator Dpysl2 (dihydropyrimidinase-like 2), and its more recently reported interaction partners LSP1 (lymphocyte specific 1), and MARCKS (myristoylated alanine rich protein kinase C substrate). Moreover, FV-DC were characterized by diminished expression of the focal adhesion proteins vinculin and talin. Attenuated expression of these cytoskeletal proteins may pinpoint on altered migratory and DC/T cell interacting properties of FV-DC.

**Fig 2:**
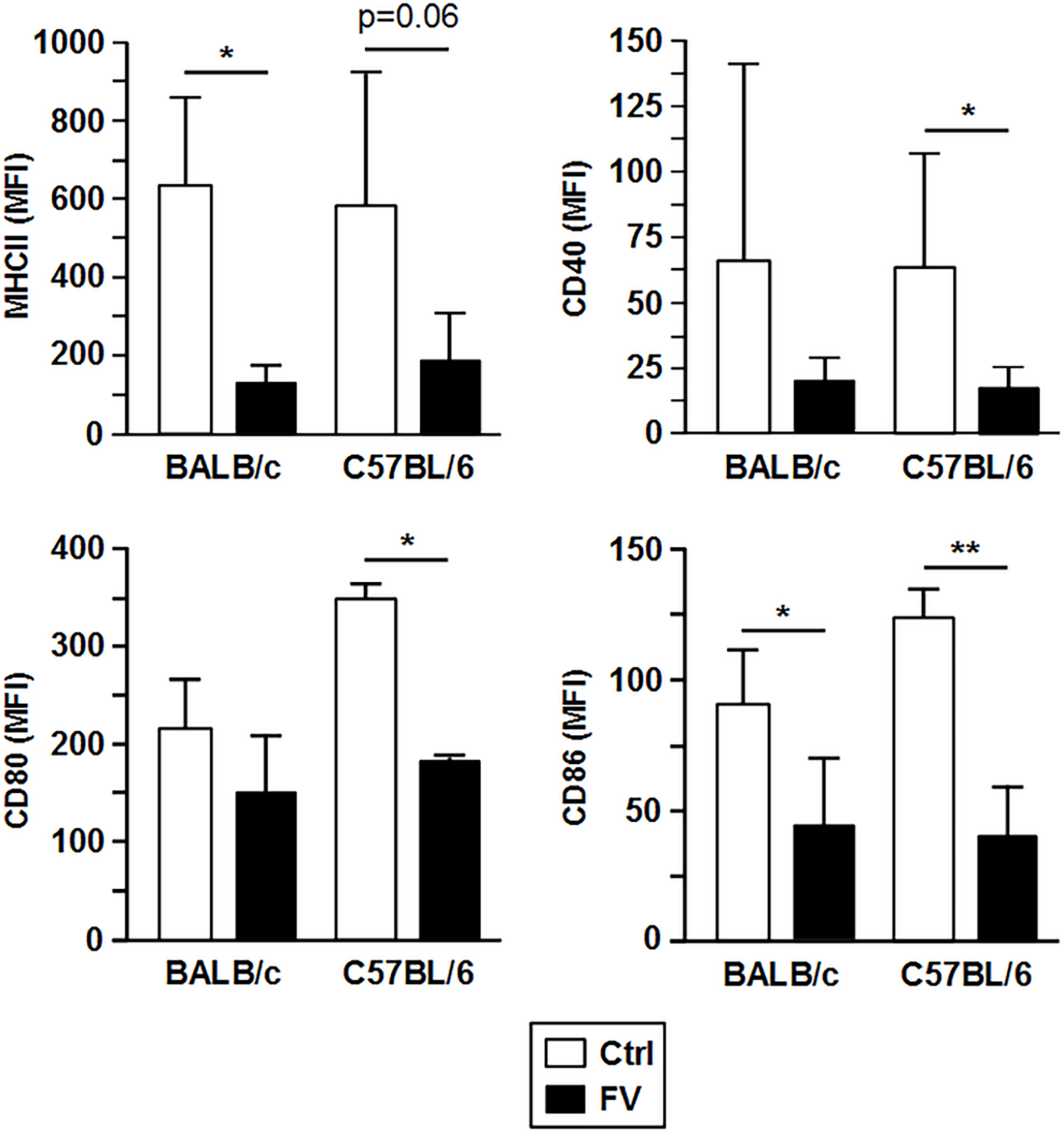
Unstimulated FV-DC are characterized by impaired expression of DC activation markers. One week after FV infection of mice (BALB/c, C57BL/6), FV-infected bone marrow cells (BM) were immuno-sorted, and differentiated to BM-DC (FV-DC) in the presence of GM-CSF (10 ng/ml). In parallel settings, BM-DC were differentiated from BM cells of untreated siblings and served as a control (Ctrl-DC). On day 6 of culture, immature DC were harvested and analysed at unstimulated state by flow cytometry for the expression of surface markers. MFI values for according surface markers of pregated CD11c^+^ cells are given. Data represent the mean±SD of 5 independent experiments (each one DC culture per group and experiment). Statistically significant differences between non-infected (Ctrl) and FV-infected (FV) BMDC (ANOVA and paired Student’s t-test) are indicated (* p< 0.05, ** p<0.01).

FV-DC contained higher contents of the lysosomal proteases Cts (cathepsin) B and D (Fig 1D) that serve to generate antigens to be loaded onto MHC-II. In addition, CtsB tailors the invariant chain that stabilizes MHC-II molecules prior to binding of antigen. In contrast, FV-DC were characterized by attenuated expression of MHC-II alleles (H2-Aa, H2-Ab1) (see also Fig 2, S4 Fig) which may limit antigen presentation to CD4^+^ T cells and thereby the induction of cellular immune responses.

### FV-infected DC of either genotype were largely non-responsive towards stimulation

Proteomic analysis largely identified FV- regulated cytoplasmic proteins. To assess the immuno-phenotype of FV-infected DC with regard to surface proteins, we performed flow cytometric analysis of FV-DC differentiated from FV-infected BM cells. In agreement with the proteome analysis, unstimulated FV-DC of either genotype were characterized by lower surface expression of MHC-II as compared with Ctrl DC (S4 Fig, left panel), although below significance in case of C57BL/6 DC (Fig 2). Activation of antigen-specific T cells requires concomittant expression of costimulatory surface proteins. We observed that FV-DC expressed less CD40, CD80, and CD86 compared to Ctrl DC in case of C57BL/6 DC. With regard to BALB/c DC, only CD86 expression was significantly impaired. No differences was found between infected DC from Balb/c versus C57/BL6 mice.

**Fig 3:**
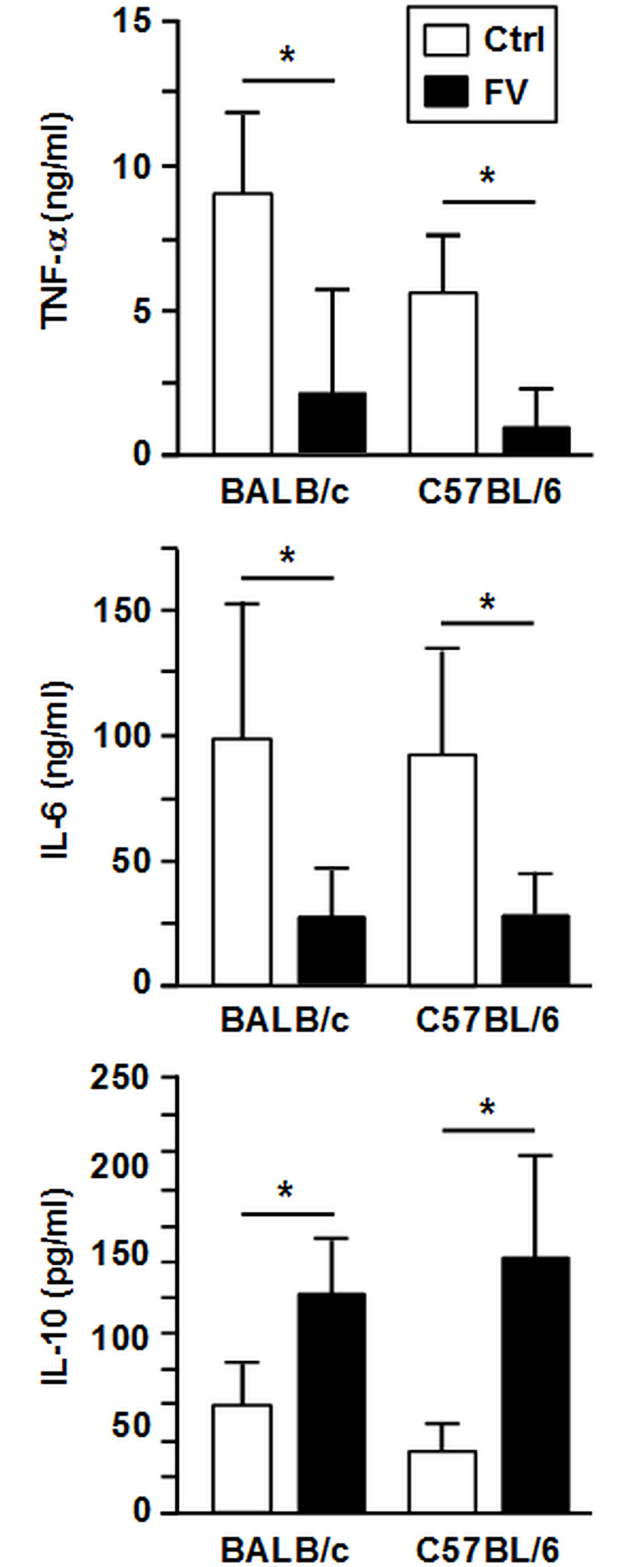
Stimulated FV-DC show attenuated expression of proinflammatory cytokines, but more IL-10. On day 6 of culture, aliquots of immature BM-DC populations derived from uninfected and FV-infected progenitor cells (see Fig 2) were harvested and stimulated over-night with LPS (100ng/ml). Cytokine contents in supernatants of stimulated BM-DC cultures were assayed by CBA. Data represent the mean±SD of 3 independent experiments (each one DC culture per group and experiment). Statistically significant differences between non-infected (Ctrl) and FV-infected (FV) BMDC (ANOVA and paired Student’s t-test) are indicated (* p<0.05).

Primary FV-infected splenic DC sorted from spleen cells of infected mice of either genotype stimulated with an immunostimulatory CpG oligonucleotide (ODN 1826) expressed lower levels of MHC-II (not significant in case of C57BL/6) and showed somewhat less expression of the costimulator CD86than Ctrl DC (S3A Fig). Similarly, FV infection also somewhat attenuated the upregulation of MHC-II and CD86 expression on BMDC after stimulation with LPS (S4 Fig). Furthermore, supernatants of LPS-stimulated FV-DC contained lower concentrations of TNF- α and of IL-6 than the corresponding Ctrl DC population, but significantly more IL-10 (Fig 3). In accordance, FV-infected and stimulated splenic DC produced more antiinflammatory IL-10 (S3B Fig). Altogether, these results suggest that FV infection of DC affects the expression of surface molecules and cytokines that are associated with their T cell stimulatory capacity, largely irrespective of their genotype.

### FV-DC exert diminished Th1/Tc1 responses

**Fig 4:**
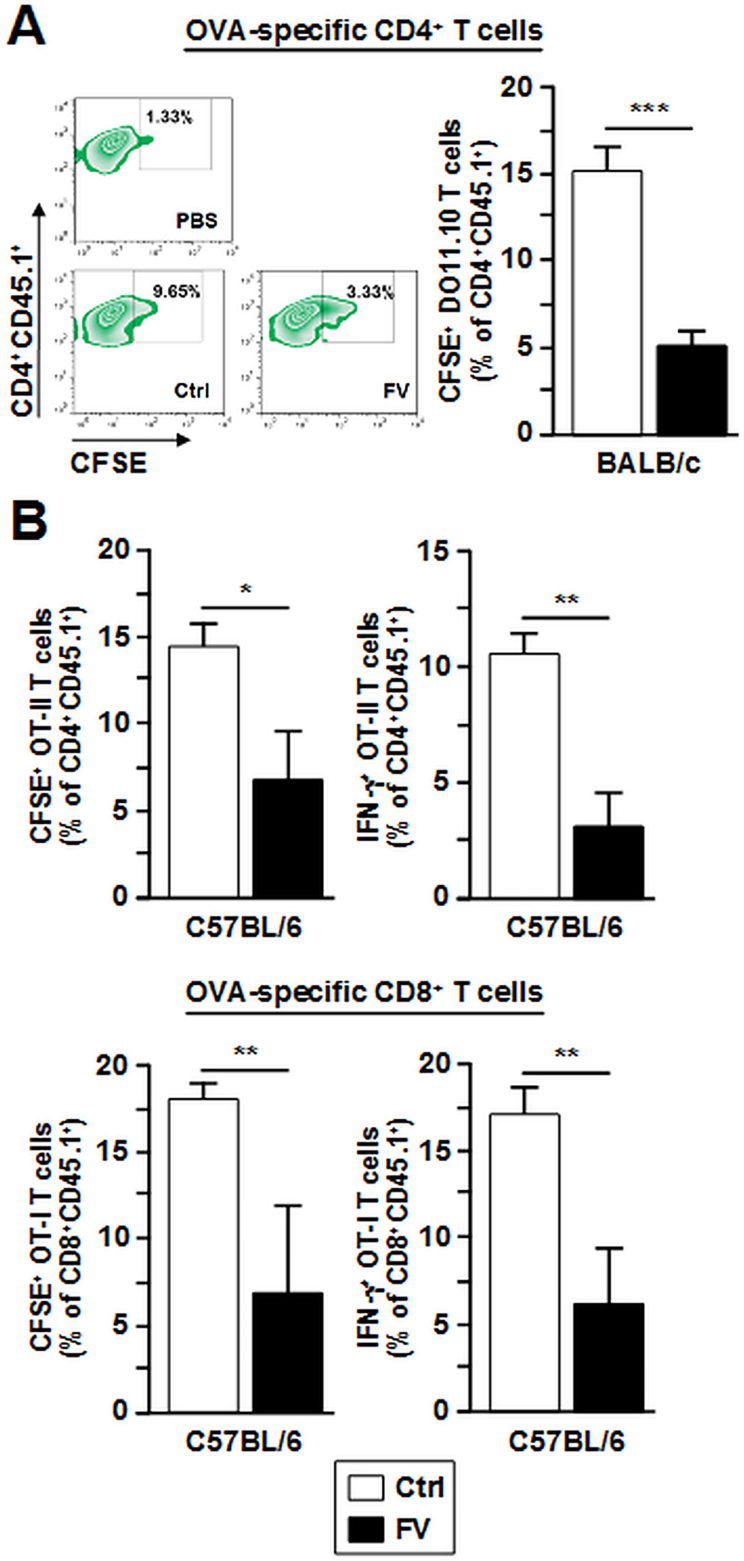
FV-DC exert impaired T cell stimulation and IFN-γ production. Isolated splenocytes (BALB/c background: DO11.10, C57BL/6 background: OT-I, OT-II; crossed with CD45.1 expressing mice of corresponding genotype) were labeled with 0.5 μmol CFSE. CFSE-labeled spleen cells (10^7^ /mouse) were injected i.v. into corresponding syngeneic BALB/c or C57BL/6 mice (each 4 mice per group). FV-DC and Ctrl-DC were generated as described (see Fig 2). DC populations were harvested on day 6 of culture and were treated over-night with LPS (100 ng/ml) and OVA peptide (each 1 μg/ml; OVA_257-264_: recognized by DO11.10 and OT-II CD4^+^ T cells, OVA_323-339_: recognized by CD8^+^ OT-I T cells). Two days after adoptive transfer of labeled spleen cells, recipients were immunized with OVA peptide-loaded and stimulated DC populations (each 3x10^6^/mouse). Non-immunized mice served as controls (PBS groups). Three days after DC transfer, mice were sacrificed and frequencies of CFSE-labeled CD45.1+ T cells within spleen cell suspensions were assessed by flow cytometry. Frequencies of in vivo proliferated (A) DO11.10 T cells, and (B, left panel) CD4^+^ OT-II T cells and CD8^+^ OT-I T cells, and (B, right panel) of according IFN-ϫ producing OT-II and OT-I T cells are given. (A, B) Data represent the mean±SD of 3 independent experiments with each 4 mice/group. Statistically significant differences between groups (ANOVA and paired Student’s t-test) are indicated (* p<0.05, ** p<0.01, *** p<0.001).

Next we determined the in vivo T cell stimulatory capacity of FV-DC. In order to study the ability of FV-infected DC to induce primary T cell responses, we used T cells (CD4^+^, CD8^+^) which respond to OVA protein derived antigens. For this, recipient mice were injected with fluorescence-labeled OVA peptide-recognizing CD4^+^ (BALB/c: DO11.10, C57BL/6: OT-II) and CD8^+^ (C57BL/6: OT-I) T cells, respectively. Afterwards, FV-DC differentiated in vitro from FV-infected BM progenitors and Ctrl DC were loaded with OVA protein, stimulated with LPS and injected as well. After 3 d, splenic T cell populations were analyzed. Antigen-loaded FV-DC of BALB/c (Fig 4A) and C57BL/6 (Fig 4B, upper left panel) genotype induced less OVA-specific CD4^+^ T cell proliferation than observed for Ctrl DC. Similarly, FV-DC of C57BL/6 background mediated less proliferation of OVA-reactive CD8^+^ T cells (Fig 4B, lower left panel). In correspondence, FV-DC induced lower frequencies of IFN- γ producing CD4^+^ and CD8^+^ T cells than Ctrl DC (Fig 4B, right panels). The reduced T cell stimulatory capacity of FV-DC was not due to limited uptake of OVA antigen since FV-DC internalized more fluorescence-labeled OVA protein than Ctrl DC (S5 Fig). Altogether, these observations are in accordance with the effects of FV infection on the DC immuno-phenotype.

### IL-10 contributes to the impaired functional state of FV-DC

**Fig 5:**
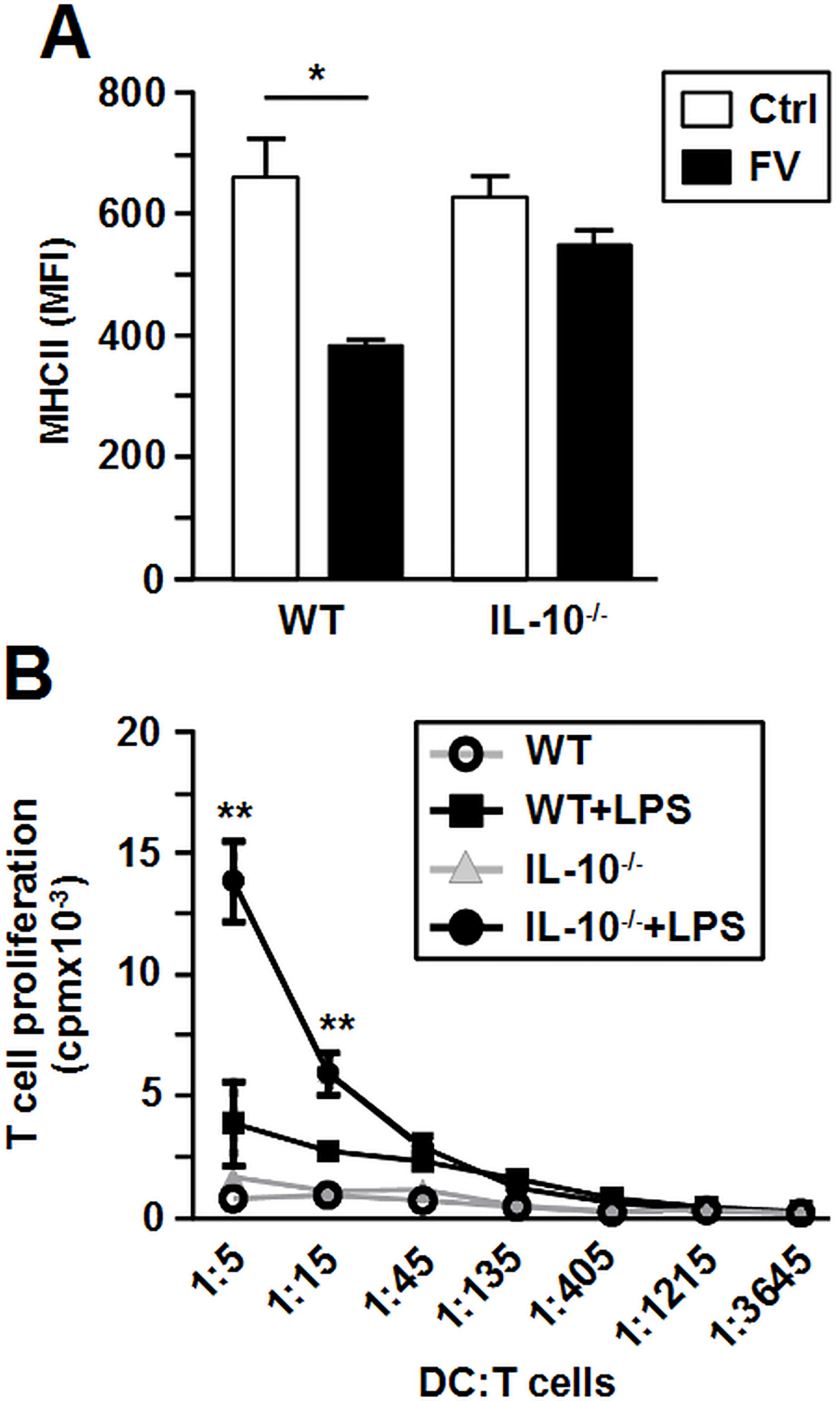
IL-10 contributes to impaired MHC-II expression and T cell stimulatory capacity of FV-DC. FV-DC derived from wild type and IL-10^-/-^ C57BL/6 mice were generated as described. Aliquots of BM-DC populations (d6; each one DC culture per group and experiment) were stimulated over-night with LPS (100 ng/ml). (A) LPS-stimulated BM-DC populations were assayed for MHC-II expression by flow cytometry. Data represent the mean MFI±SD of 3 independent experiments. (B) T cell proliferation as evoked by FV-infected BM-DC populations was assessed by uptake of ^3^H-thymidine during the last 16 h of culture. Data represent mean±SD of triplicates. Results from one representative out of 3 independent experiments are shown. (A,B) Statistically significant differences (A) between non-infected (Ctrl) and FV-infected (FV) BMDC of same genotype, and (B) between stimulated versus unstimulated groups of same genotype are indicated (* p<0.05, ** p<0.01) (ANOVA and paired Student’s t-test).

IL-10 is a potent anti-inflammatory cytokine known to affect the activation state of antigen presenting cells, including DC. Since LPS-stimulated FV-DC produced IL-10 at elevated extent we analyzed whether this contributed to their reduced T cell stimulatory capacity. To this end, IL-10^-/-^ mice (C57BL/6) were infected with FV, and FV-DC derived from immune-magnetically sorted infected BM progenitor cells were analyzed. LPS-stimulated Ctrl DC of wild type (WT) and IL-10^-/-^ mice expressed MHC-II at comparable levels (Fig 5A). LPS-stimulated FV-DC of WT genotype showed impaired MHC-II expression, while IL-10^-/-^ FV-DC retained stimulation-induced upregulation of MHC-II expression. OVA-loaded and LPS-stimulated WT FV-DC poorly activated OVA-reactive CD4^+^ T cells in vitro, similar to unstimulated FV-DC of either genotype (Fig 5B). In contrast, OVA-presenting IL-10^-/-^ FV-DC induced much stronger CD4^+^ T cell proliferation after LPS stimulation than at unstimulated state (statistically significant at the two highest DC:T cell ratios). These findings suggest that FV-induced IL-10 negatively regulates MHC-II expression on LPS-stimulated FV-DC and consequently their T cell stimulatory capacity.

### FV-DC induce Th2-biased responses

**Fig 6:**
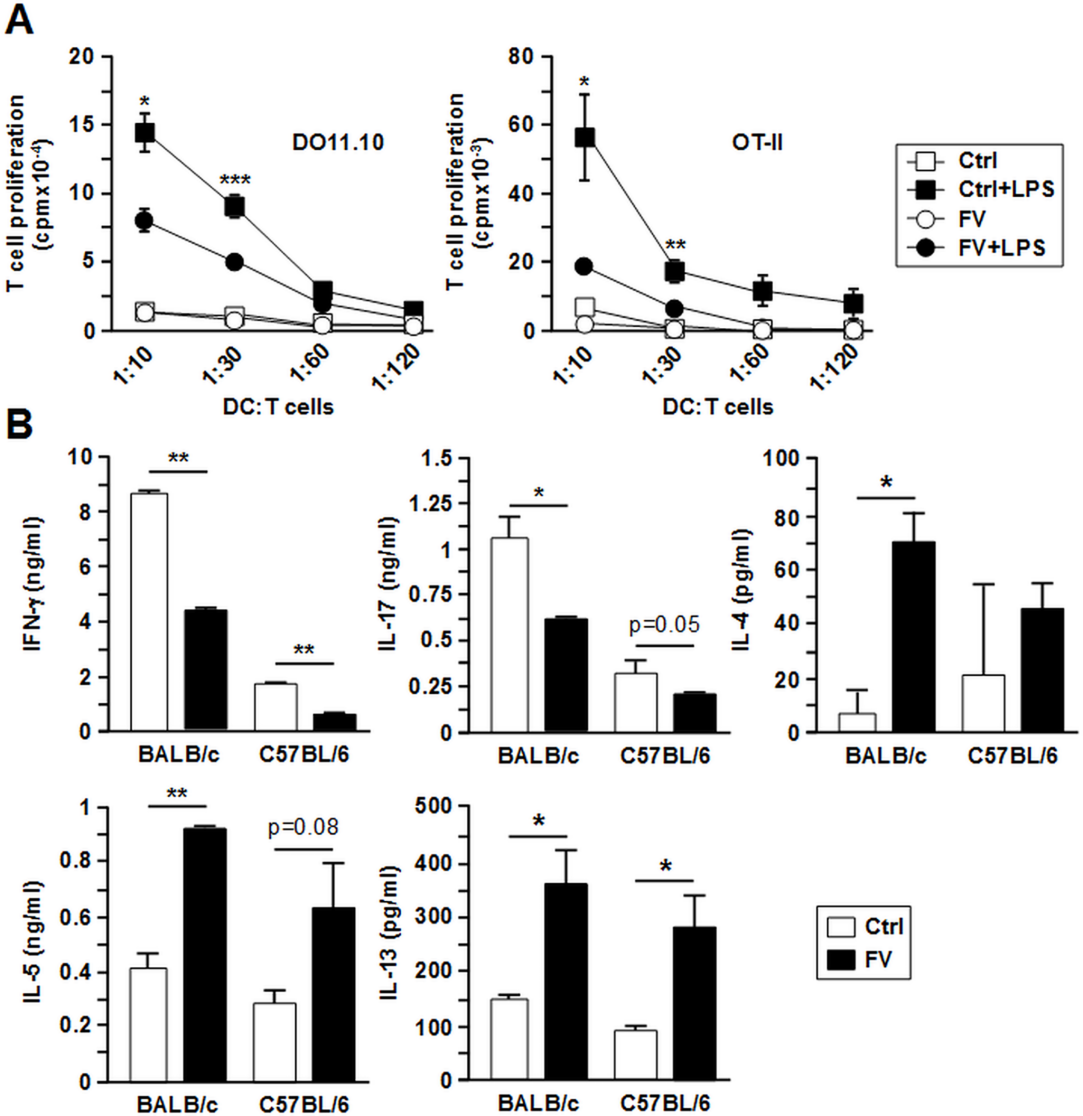
FV-infected DC favor Th2 responses. BM-DC populations (d6) were loaded with OVA_257-264_ peptide (1 μg/ml), and aliquots were stimulated with LPS (100ng/ml). On the next day, titrated numbers of BM-DC (each one BMDC culture per group and experiment) were cocultured with OVA peptide-specific CD4^+^ T cells (DO11.10, BALB/c; each 5x10^4^/well) in triplicates for 72 h. (A) T cell proliferation was assessed by uptake of ^3^H-thymidine for the last 16 h of culture. Data represent mean±SD of triplicates. Results from one representative out of 3 independent experiments are shown. (B) Cytokine contents in supernatants of DC/T cell cocultures that harbored LPS-stimulated DC (ratio 1:10) were assayed by CBA. Data represent the mean±SD of 3 independent experiments each. (A,B) Statistically significant differences (A) between stimulated versus unstimulated groups of same genotype, and (B) between groups containing non-infected (Ctrl) and FV-infected (FV) BMDC of same genotype groups are indicated (* p<0.05, ** p<0.01, *** p<0.001) (ANOVA and paired Student’s t-test).

Besides the magnitude of T cell stimulation, the polarization of activated T cells determines the efficacy of pathogen clearance. To assess the cytokine pattern of CD4^+^ T cells that were stimulated by FV-infected DCs, OVA-loaded BM-DC were co-cultured with OVA-reactive CD4^+^ T cells in vitro. In general, unstimulated DC evoked no major CD4^+^ T cell proliferation (Fig 6A). As expected, LPS-stimulated FV-DC of either genotype did not induce stronger T cell proliferation in contrast to the corresponding group of Ctrl DC (statistically significant at the two highest DC:T cell ratios). DC/T cell co-cultures that contained LPS-stimulated FV-DC contained lower levels of Th1/Th17-associated IFN- γ and IL-17 (not significant in case of BALB/c DC) in the supernatant than measured in cocultures containing LPS-stimulated Ctrl DC. In contrast, in cocultures containing LPS-stimulated FV-DC significantly higher levels of the Th2-associated cytokines IL-4 and IL-5 (not significant in case of C57BL/6) and IL-13 were observed (Fig 6B). These findings indicate that FV-DC may favor Th2 polarization as reflected by elevated levels of Th2 cytokines (esp. IL-13) and attenuated Th1 cytokine (IFN- γ) production.

Taken together, our study demonstrates that FV infection induces profound alterations of the DC proteome, which may affect the induction of Th1/Tc1-driven adaptive anti-viral immune responses in a mouse genotype-independent manner. FV-induced IL-10 in infected DC after stimulation with LPS contributed to the impaired immunophenotype and consequently attenuated the T cell stimulatory activity of FV-DC. In addition, FV-DC favored Th2 responses, and both factors may contribute to immune evasion of FV.

## Discussion

We showed previously that FV infects DC of BALB/c origin which results in the release of viral particles, and the induction of CD4^+^ regulatory T cells [16], suggesting that FV directly affects DC to prevent the induction of a sustained anti-FV adaptive immune response. In this study, we analyzed in more detail the effects of FV infection on the DC proteome and immunophenotype to understand how FV may limit the induction of DC-dependent adaptive immune responses.

We identified a number of host proteins that were concurrently regulated in FV-DC of distinct genotypes, and therefore may play a role in functional reprogramming of DC to promote viral immune-evasion. FV-induced down-regulation of the focal adhesion proteins vinculin (Vcl) and talin (Tln1) is reminiscent of previous according findings on HIV-1 and MMLV infected cells [31]. Impaired expression of actin-binding proteins by FV-DC (see Fig 1C) may contribute to virion transport into the nucleus, and may support retroviral replication and capsid formation [32].

Moreover, we and others have shown that dynamic cytoskeletal rearrangements are essential for DC migration and the formation and integrity of the immunological synapse [33, 34]. Altogether, FV-induced alterations of cytoskeletal protein expression may on one hand serve to support the FV life cycle, but also diminish the antigen presenting function of DC.

Productive infection of DC with FV resulted in gross alterations of their metabolome in a genotype-independent manner. Several viruses are known to establish a metabolic state to fuel virion production which is similar to rapidly proliferating malignant cells, termed "Warburg effect" [35]. By this, cells ferment glucose into lactate even under aerobic conditions to yield both ATP and metabolite precursors [36]. This metabolic pathway has been shown to contribute to immunosuppression in the tumor microenvironment and is associated with worse prognosis [37], and it may also be employed by FV to facilitate immune evasion. Hbb-b2 was shown to interact with several mitochondiral proteins including ATPsynthase subunits, and was attributed to play a role in mitochondrial respiration [38].

Our finding of enhanced levels of Nceh1 which is largely responsible for cholesterol hydrolysis in macrophages [39], and reduced contents of EHD1 as required for intracellular cholesterol storage [40] suggests FV-induced attenuation of cellular cholesterol levels which in turn may negatively affect the frequency of MHCII receptors that cluster within cholesterol-containing microdomains [41].Moreoever, attenuated MHCII expression by FV-DC was associated with elevated levels of the cathepsins CtsD and CtsB which contribute to intracellular protein turnover [42]. More recently, CtsB was reported to negatively regulate MHC-II expression [43]. Furthermore, in HIV-infected cells, upregulated CtsB was required for processing of nascent HIV particles since its knockdown resulted in an accumulation of virus particles in autophagosomes [44].

We also investigated surface molecule expression, cytokine production and T cell stimulatory capacity of FV-DC. We observed enhanced IL-10 production in LPS-stimulated FV-DC which in turn limited MHCII expression by DC, and attenuated their CD4^+^ T cell stimulatory capacity. In agreement, exogenous IL-10 is well known to induce a maturation-resistant state in DC [45]. In agreement, FV-DC of either genotype expressed costimulatory molecules required for the activation of CD4^+^ and CD8^+^ T cells at lower extent as compared with non-infected DC. Elevated IL-10 production is a common characteristic of virus-infected antigen presenting cells, and is accompanied by diminished production of Th1/Tc1 inducing proinflammatory cytokines [46]. While some viruses encode for viral IL-10 [47], others modulate IL-10 expression of the host cell [48]. Preferential integration of SFFV provirus into the Spi1 (SFFV proviral integration oncogene) gene locus [49] that encodes Spi1/PU.1, and of F-MuLV provirus into the Fli-1 (Friend leukemia integration 1) gene [50] yields elevated expression of the corresponding target genes. PU.1 and Fli-1 are members of the Ets transcription factor family and may cooperate to modulate target gene expression [51]. In agreement with productive FV infection of DC [16], we observed enhanced Fli-1 and PU.1 mRNA levels in FV-DC as compared with Ctrl DC (data not shown). Fli-1 was shown to trans-activate IL-10 in murine myeloid cells [52]. For human IL-10, PU.1 was demonstrated to bind within the distal promoter region [53]. This binding site is evolutionarily conserved between human and mouse. Further studies are necessary to delineate the mechanism of FV-mediated IL-10 induction, and the overall role of this cytokine for the altered immuno-phenotype and Th2-promoting properties of FV-infected DC.

Besides autocrine inhibitory effects, FV-DC derived IL-10 in a paracrine manner may also inhibit T cell proliferation [54] and IFN- γ production by T cells [55]. Therefore, the ability of FV to impair the Th1/Tc1 polarizing capacity of infected DC may constitute an important immuno-evasive mechanism of the virus. Indeed, we have previously demonstrated that in vivo neutralization of IFN- γ enhanced the susceptibility of C57BL/6 mice towards FV infection [5]. We also observed elevated concentrations of Th2-associated cytokines (IL-4, IL-5, IL-13) in cocultures of FV-DC of either genotype with antigen-specific CD4^+^ T cells. This finding indicates that the well known strain-specific differences between C57BL/6 and BALB/c in mounting Th1 vs. Th2 responses do not contribute to differences in the disease course of FV infection in these strains. FV-induced Th2-skewing of DC-dependent T cell responses may be explained in part by elevated IL-10 due to its inhibitory effects on Th1-inducing cytokines [56], and is in accordance with our previous finding of impaired production of IL-12 by FV-infected BALB/c DC [16]. For other viruses Th2-skewing properties to prevent efficient Th1/Tc1 immune responses as an important immuno-evasive mechanism have been described as well [57].

Other FV-induced mediators may contribute to the Th2-promoting capacity of FV-DC as well. In this regard, proteome analysis identified Alox5 as upregulated in FV-DC. Alox5 mediates the first, rate-limiting step of leukotriene B4 (LTB4) generation [58]. LTB4 is generated by myeloid cells and is elevated in response to pathogen-derived stimuli. LTB4 acts as a chemo-attractant for neutrophils and other leukocytes, and was shown to exert anti-viral and anti-bacterial effects, in part by mediating release of antimicrobial peptides [59]. However, LTB4 preferentially attracts Th2 cells, which in turn produce Th2-associated chemokines, and thereby may amplify Th2 responses at the site of inflammation [60]. In accordance, Alox5^-/-^ mice infected with *Brucella abortus* generated a more pronounced Th1 response than wild type mice [61]. Further studies are required to elucidate the potential contribution of Alox5 to the Th2-promoting capacity of FV-DC.

As shown above, we did not observe major genotype-specific differences between FV-DC with regard to their immuno-phenotype and T cell stimulatory porperties. In addition, several important factors like genotype-specific expression of Apobec-3 isoforms [10], and the differential ability of MHCII alles to present immunodominant FV antigens [11] were demonstrated as essential for the outcome of FV infection. However, we can not rule out that some of the genotype-specifically FV-regulated proteins as identified in our study (see S1 File) may contribute to the chronic (C57BL/6) versus fatal (BALB/c) course of FV infection in vivo. Hence, more studies are necessary to analyze the role of these proteins for DC to modulate FV infection, especially in C57BL/6 mice which develop a chronic infection.

In conclusion, FV-DC in a genotype-independent manner show similar impairment of their immunophenotype and T cell stimulatory potential, and a pronounced Th2-skewing phenotype. FV-induced IL-10 may play an important role in these regards. Moreover, dysregulated expression of metabolic, cytoskeletal and other proteins in FV-DC may support FV virion generation and the functional impairment of DC.

## Supporting information

S1 Fig Gating strategy for flow cytometric analysis of DC. (1) Cell debris was excluded. (2) Cell doublets were excluded. (3) CD11c^+^ cells were gated. (4) CD11c^+^ cells were further analyzed for expression of MHCII, CD40, CD80 and CD86 (gated).
(TIF)

S2 Fig Infection of BALB/c mice with FV yields higher frequencies of infected cells. Mice were inoculated i.v. with FV (BALB/c: 3,000 IU, C57BL/6: 10,000 IU). (A) After one week, (A) CD11c^+^ splenic DC and (B) bone marrow cells were immuno-sorted using a FV p34-specific antibody. The frequencies of FV-infected cells were assessed by FACS analysis. Data represent the mean±SEMD of (A) 5 and (B) 15 mice. (A,B) Statistical significant differences between groups are indicated (* p<0.05, ** p<0.01).
(TIF)

S3 Fig FV-infected splenic DC fail to acquire a stimulation-induced mature immuno-phenotype, accompanied by elevated IL-10. Mice (BALB/c, C57BL/6) were inoculated i.v. with FV (BALB/c: 3,000 IU, C57BL/6: 10,000 IU). After one week, FV-infected CD11c^+^ splenic DC were immuno-sorted using a FV p34-specific antibody from FV-infected mice. CD11^+^ splenic DC from healthy siblings served as controls (Ctrl). Isolated splenic DC were stimulated over-night with LPS (100 ng/ml). (A) Expression of surface markers (CD11c, MHCII, CD86) of uninfected (Ctrl) and FV-infected (FV) splenic DC was assayed by flow cytometry. *Left panel*: Dot bplots of co-detected CD11c and CD86 are representative of 5 experiments each. Frequencies of subpopulations are indicated. *Right panel*: Mean fluorescence intensities (MFI) of MHCII and CD86 on CD11c^+^ DC populations are given (B) Culture supernatants of stimulated splenic DC cultures were analyzed for IL-10 by CBA. (A, *right panel*, and B) Data represent the mean±SD of 5 independent experiments each. Statistically significant differences between groups are indicated (* p<0.05, ** p<0.01).
(TIF)

S4 Fig LPS-stimulated FV-DC show a tendency to upregulate MHCII and CD86 at lower extent. On day 6 of culture, aliquots of immature BM-DC populations derived from uninfected and FV-infected progenitor cells (see Fig 2) were harvested and stimulated over-night with LPS (100ng/ml). Expression of surface markers (CD11c, MHC-II, CD86) of unstimulated and LPS-stimulated C57BL/6 DC populations (Ctrl, FV) was assayed by flow cytometry. Dot plots are representative of 3 experiments each. Frequencies of subpopulations are indicated.
(TIF)

S5 Fig FV-DC are characterized by enhanced endocytic activity. On day 6 of culture, aliquots of immature BMDC populations derived from uninfected (Ctrl) and FV-infected progenitor cells of BALB/c and C57BL/6 mice (see Fig 2) were harvested and incubated (2.5x10^5^ per sample) in duplicates with FITC-labeled OVA (1 mg/ml) obtained from Life Technologies in parallel at 37°C and 4°C as a control. After the indicated periods of time endocytic uptake of FITC-OVA was stopped, cells were stained with APC-labeled anti-CD11c specific antibody, and samples were analyzed by flow cytometry. Data represent the mean±SEMD of 5 independent experiments (each one BMDC population per group and experiment). Statistically significant differences between groups are indicated (* p<0.05, ** p<0.01, *** p<0.001).
(TIF)

S1 File FV-regulated proteins in DC. (XLS)

S2 File Enrichment analysis of FV-regulated proteins. (XLS)
