# Supplementary figures and images for: Friend virus limits adaptive cellular immune responses by imprinting a maturation-resistant and T helper type 2-biased immunophenotype in dendritic cells
Source: PLoS One. 2018 Feb 9;13(2):e0192541. doi: 10.1371/journal.pone.0192541 (PMC5806892; doi:10.1371/journal.pone.0192541)

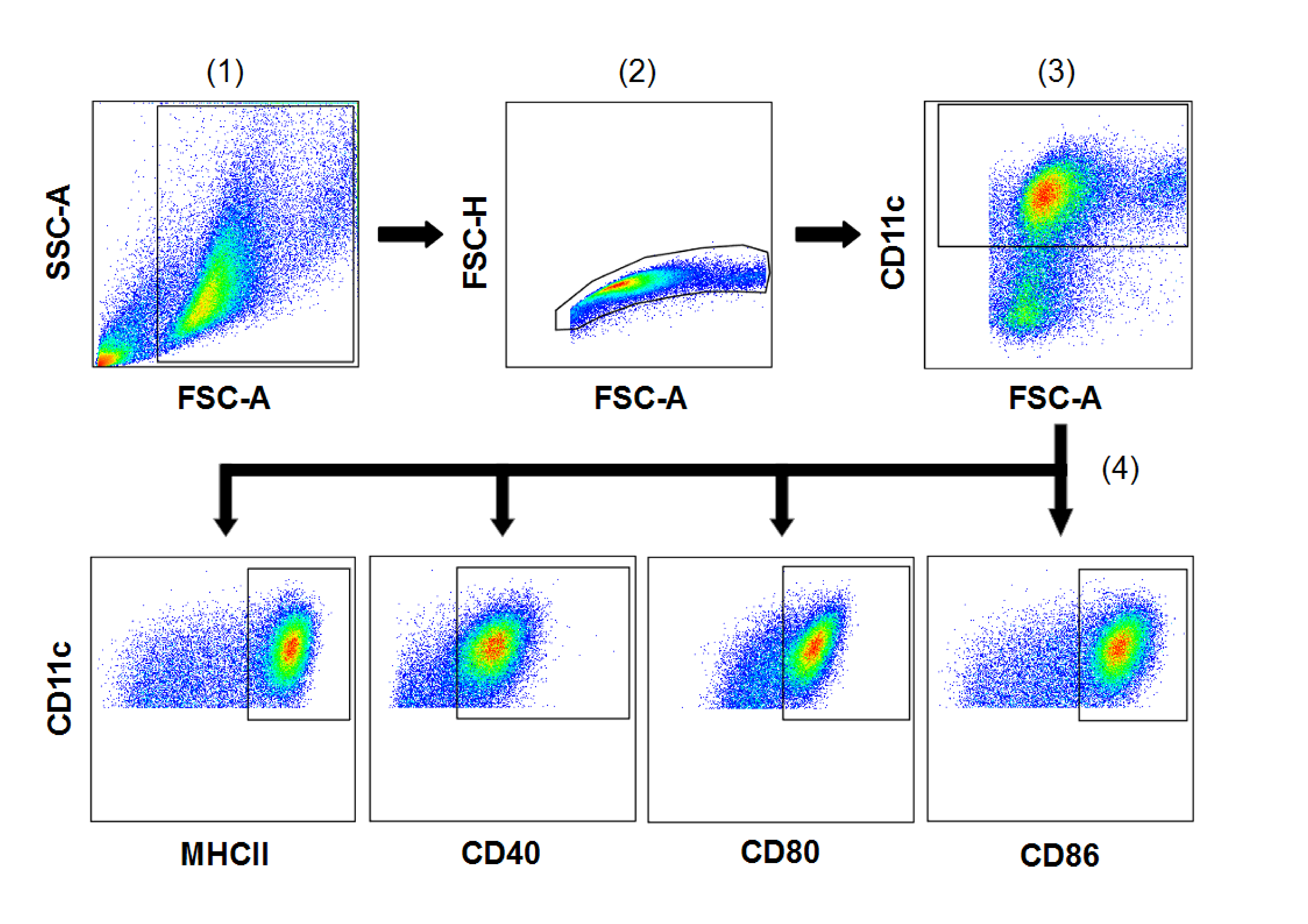

Supplement: S1 Fig — (1) Cell debris was excluded. (2) Cell doublets were excluded. (3) CD11c+ cells were gated. (4) CD11c+ cells were further analyzed for expression of MHCII, CD40, CD80 and CD86 (gated). (TIF) [file pone.0192541.s001.tif]

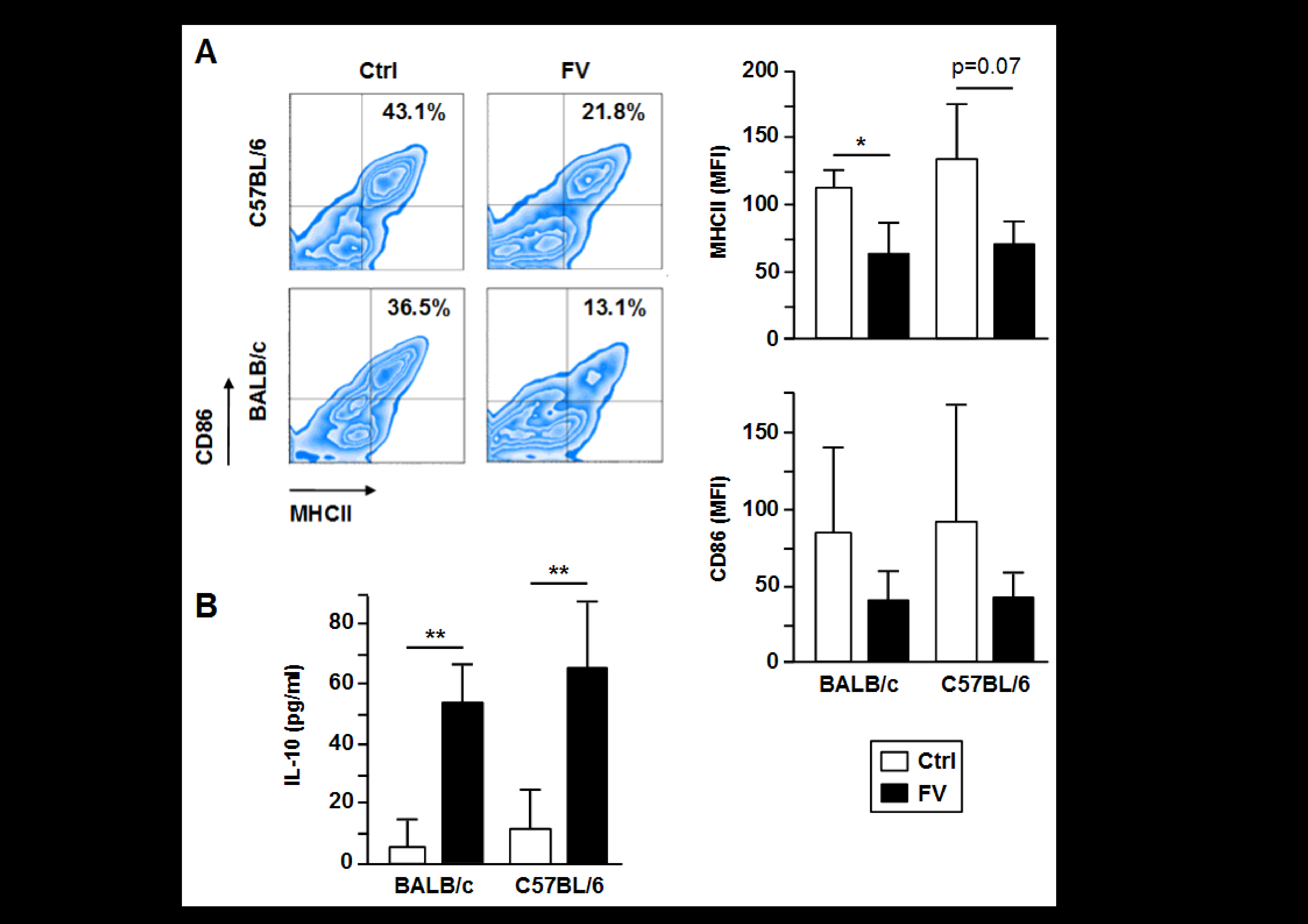

Supplement: S3 Fig — Mice (BALB/c, C57BL/6) were inoculated i.v. with FV (BALB/c: 3,000 IU, C57BL/6: 10,000 IU). After one week, FV-infected CD11c+ splenic DC were immuno-sorted using a FV p34-specific antibody from FV-infected mice. CD11+ splenic DC from healthy siblings served as controls (Ctrl). Isolated splenic DC were stimulated over-night with LPS (100 ng/ml). (A) Expression of surface markers (CD11c, MHCII, CD86) of uninfected (Ctrl) and FV-infected (FV) splenic DC was assayed by flow cytometry. Left panel: Dot bplots of co-detected CD11c and CD86 are representative of 5 experiments each. Frequencies of subpopulations are indicated. Right panel: Mean fluorescence intensities (MFI) of MHCII and CD86 on CD11c+ DC populations are given (B) Culture supernatants of stimulated splenic DC cultures were analyzed for IL-10 by CBA. (A, right panel, and B) Data represent the mean±SD of 5 independent experiments each. Statistically significant differences between groups are indicated (* p<0.05, ** p<0.01). (TIF) [file pone.0192541.s003.tif]

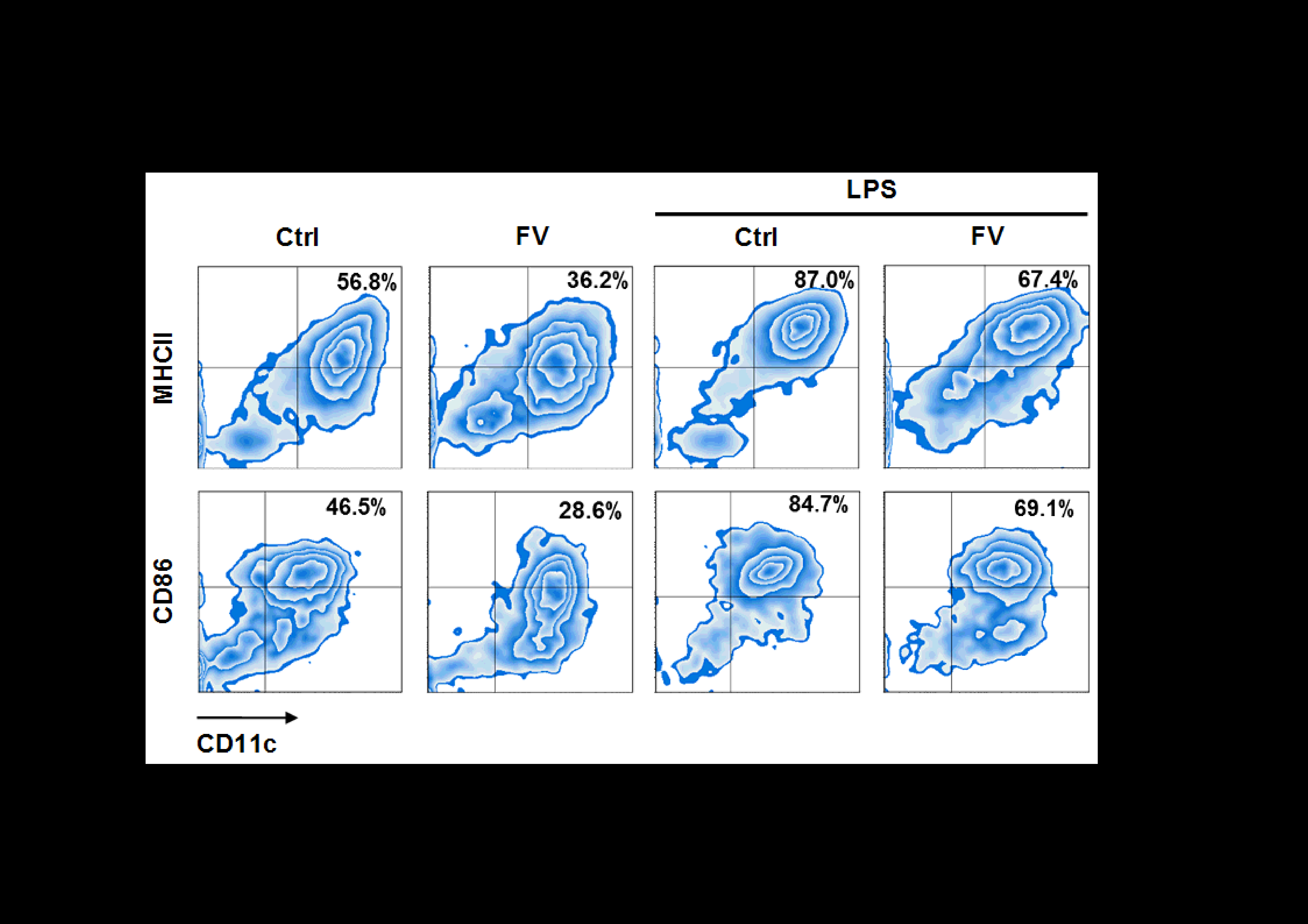

Supplement: S4 Fig — On day 6 of culture, aliquots of immature BM-DC populations derived from uninfected and FV-infected progenitor cells (see Fig 2) were harvested and stimulated over-night with LPS (100ng/ml). Expression of surface markers (CD11c, MHC-II, CD86) of unstimulated and LPS-stimulated C57BL/6 DC populations (Ctrl, FV) was assayed by flow cytometry. Dot plots are representative of 3 experiments each. Frequencies of subpopulations are indicated. (TIF) [file pone.0192541.s004.tif]
